# Analytical Strategies for Tocopherols in Vegetable Oils: Advances in Extraction and Detection

**DOI:** 10.3390/ph18081137

**Published:** 2025-07-30

**Authors:** Yingfei Liu, Mengyuan Lv, Yuyang Wang, Jinchao Wei, Di Chen

**Affiliations:** 1 Zhengzhou Research Base, National Key Laboratory of Cotton Bio-Breeding and Integrated Utilization, School of Pharmaceutical Sciences, Zhengzhou University, Zhengzhou 450001, China; 2 Macau Centre for Research and Development in Chinese Medicine, State Key Laboratory of Quality Research in Chinese Medicine, Institute of Chinese Medical Sciences, University of Macau, Macao SAR 999078, China

**Keywords:** tocopherols, vitamin E, vegetable oils, extraction techniques, analytical methods

## Abstract

Tocopherols, major lipid-soluble components of vitamin E, are essential natural products with significant nutritional and pharmacological value. Their structural diversity and uneven distribution across vegetable oils require accurate analytical strategies for compositional profiling, quality control, and authenticity verification, amid concerns over food fraud and regulatory demands. Analytical challenges, such as matrix effects in complex oils and the cost trade-offs of green extraction methods, complicate these processes. This review examines recent advances in tocopherol analysis, focusing on extraction and detection techniques. Green methods like supercritical fluid extraction and deep eutectic solvents offer selectivity and sustainability, though they are costlier than traditional approaches. On the analytical side, hyphenated techniques such as supercritical fluid chromatography-mass spectrometry (SFC-MS) achieve detection limits as low as 0.05 ng/mL, improving sensitivity in complex matrices. Liquid chromatography-tandem mass spectrometry (LC-MS/MS) provides robust analysis, while spectroscopic and electrochemical sensors offer rapid, cost-effective alternatives for high-throughput screening. The integration of chemometric tools and miniaturized systems supports scalable workflows. Looking ahead, the incorporation of Artificial Intelligence (AI) in oil authentication has the potential to enhance the accuracy and efficiency of future analyses. These innovations could improve our understanding of tocopherol compositions in vegetable oils, supporting more reliable assessments of nutritional value and product authenticity.

## 1. Introduction

Tocopherols, as vital constituents of vitamin E, are lipid-soluble antioxidants widely recognized for their role in maintaining human health and enhancing the oxidative stability of vegetable oils [1,2]. As naturally occurring compounds with pharmacological relevance, tocopherols are increasingly studied not only as nutritional markers but also as potential bioactive agents in drug development [3,4]. Their structural diversity—comprising α-, β-, γ-, and δ-isomers—varies with the botanical origin and processing conditions, affecting both functional properties and therapeutic potential.

Pharmacologically, tocopherols exhibit potent antioxidant properties, which help mitigate the oxidative stress associated with chronic diseases, such as cardiovascular disease, diabetes, and cancer [5]. They also demonstrate anti-inflammatory and neuroprotective effects, with potential in preventing neurodegenerative disorders like Alzheimer’s and Parkinson’s [6,7]. Due to these therapeutic benefits, tocopherols are increasingly incorporated into dietary supplements, therapeutic formulations, and cosmeceuticals.

Given the growing interest in the physiological and pharmacological roles of tocopherols, the demand for accurate and efficient analytical techniques has substantially increased. Currently, a variety of methods are employed to meet these demands, including robust high-performance liquid chromatography with ultraviolet detection (HPLC-UV) [8] and highly sensitive and specific HPLC coupled with mass spectrometry (HPLC-MS) [9,10]. In particular, when combined with multiple reaction monitoring (MRM) acquisition modes, HPLC-MS enables the precise quantification of tocopherol isomers. However, due to the complex nature of lipid-rich matrices (such as fatty acids, phospholipids, and other lipophilic compounds), effective sample cleanup is often required to ensure compatibility with chromatographic systems [11]. To address this, a range of selective extraction techniques have been developed, including liquid–liquid extraction (LLE) [12] and solid-phase extraction (SPE) [13,14], along with their various modifications. In pursuit of environmental sustainability, green extraction approaches—such as supercritical fluid extraction and the use of deep eutectic solvents—have also been employed [15]. Moreover, spectroscopic and electrochemical methods are being explored for rapid and cost-effective screening, showing considerable promise for high-throughput applications and on-site testing scenarios [16].

**Figure 1:**
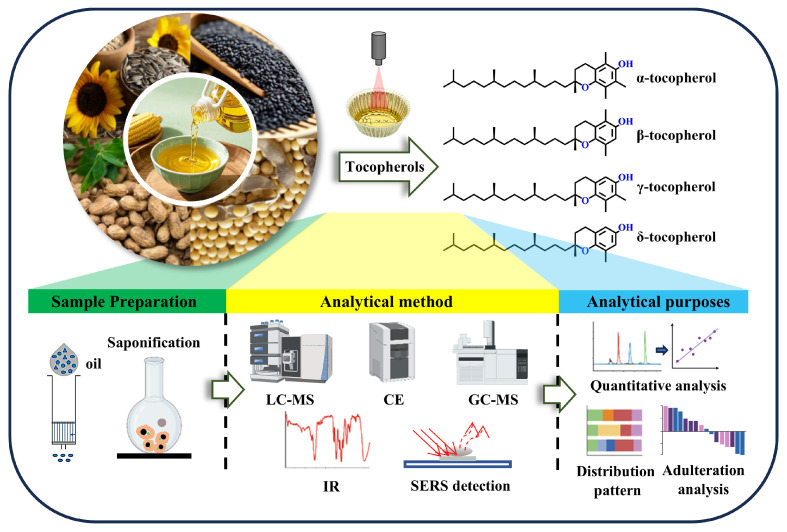
Overview of the sample preparation and analytical detection of tocopherols in vegetable oils.

This review systematically summarizes recent progress in tocopherol extraction and analytical technologies, focusing on key advances in chromatographic, mass spectrometric, spectroscopic, and electrochemical methods. Special emphasis is placed on improvements in extraction sustainability, analytical performance, and practical applicability (Figure 1). This review also highlights the potential of these techniques to support the quality control, authenticity verification, and compositional profiling of tocopherols in vegetable oils. Compared to existing reviews, which mainly cover the chemistry, metabolism, and extraction methods of vitamin E, this article provides a more targeted discussion on the latest analytical techniques, particularly in the context of sustainability and real-world applicability [17,18]. Future directions—such as method integration, miniaturization, and automation—are discussed in the context of meeting the increasing demand for precise, efficient, and environmentally sustainable analytical solutions.

## 2. Physicochemical Characteristics and Occurrence of Tocopherols in Vegetable Oils

### 2.1. Structure and Physicochemical Properties

**Figure 2:**
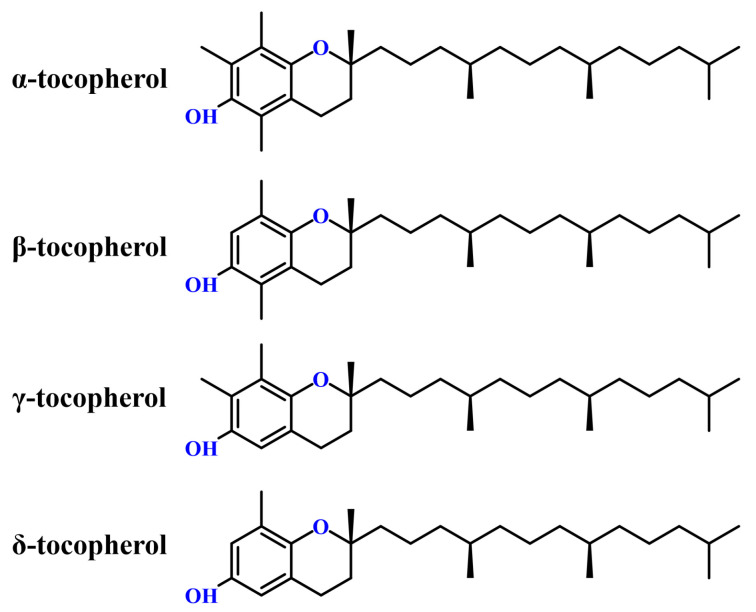
Chemical structures of α-, β-, γ-, and δ-tocopherols.

Tocopherols are a class of lipophilic phenolic compounds belonging to the vitamin E family, characterized by a chromanol ring and a phytyl-derived saturated side chain. They exist in four naturally occurring homologues—α, β, γ, and δ—distinguished by the number and position of methyl groups on the chromanol ring (Figure 2) [19,20]. Among these, α-tocopherol exhibits the highest vitamin E activity in humans due to its stereochemistry and preferential binding affinity to α-tocopherol transfer protein (α-TTP) in the liver [21]. The phenolic hydroxyl group in the chromanol ring confers antioxidant properties by donating hydrogen atoms to neutralize reactive oxygen species (ROS), while the hydrophobic phytyl chain ensures integration into lipid-rich environments such as cell membranes and vegetable oils [22,23]. These amphiphilic features influence the physicochemical behavior of tocopherols, including solubility, stability, and interactions with extraction solvents.

Furthermore, structural variations among tocopherol homologues affect their polarity and retention behavior during chromatographic separation. For example, δ-tocopherol, with fewer methyl substituents on the chromanol ring compared to other tocopherol homologues, is more polar, which affects its co-elution behavior and retention times in chromatographic columns [8,24]. A clear understanding of these structure–property relationships is essential for interpreting the behavior of tocopherols in the extraction and detection systems discussed in later sections.

### 2.2. Occurrence and Distribution in Vegetable Oils

Tocopherols are ubiquitously present in plant-derived oils, but their concentration and homolog composition vary significantly depending on the botanical origin, extraction method, and degree of oil refinement. Generally, γ-tocopherol tends to be the principal form in oils such as soybean, corn, and sesame, whereas α-tocopherol is more prominent in oils like sunflower and olive [25,26]. Yuan et al. also compared the total tocopherol content across various vegetable oils, revealing that soybean oil had the highest level (39.9 mg/100 g), followed by corn oil (36.06 mg/100 g), olive oil (29.42 mg/100 g), and camellia oil (17.72 mg/100 g) [27].

These compositional differences are important not only for assessing the nutritional value and oxidative stability of oils but also for verifying their authenticity. The distribution of tocopherol isomers can serve as a chemotaxonomic marker or indicator of adulteration. For instance, a high γ-/α-tocopherol ratio may suggest the adulteration of olive oil with lower-cost oils such as soybean or corn oil [28,29].

Refinement processes such as bleaching and deodorization can substantially reduce the native tocopherol content, thereby influencing both the antioxidant capacity of the final product and the detectability of minor components [30,31,32]. Therefore, a precise understanding of tocopherol distribution across various oil matrices is essential for developing sensitive and selective analytical methods tailored to specific oil types, quality control requirements, and the optimization of production processes.

## 3. Sample Preparation for Tocopherols in Vegetable Oils

### 3.1. Direct Dilution and Saponification Strategies

Given the complexity of oily matrices, appropriate sample preparation is critical for the efficient extraction and accurate quantification of tocopherols in vegetable oils. In certain studies, oils are directly diluted with organic solvents prior to chromatographic analysis, thereby bypassing traditional extraction or saponification procedures [8,33,34,35]. This simplified approach accelerates sample throughput and reduces the risk of analyte loss or degradation during handling.

Direct dilution using solvents, such as n-hexane, is widely adopted due to its compatibility with the lipophilic nature of tocopherols and non-polar mobile phases in normal-phase chromatography, where the stationary phase is typically polar (e.g., silica gel), which facilitates the separation of lipophilic compounds [8,33,34]. Alternatively, solvent mixtures such as acetonitrile and tetrahydrofuran are used to accommodate reversed-phase chromatographic analysis, which is more commonly applied [28]. Although these simplified methods are well suited for routine analysis, they lack matrix cleanup steps, which can lead to several issues. Coexisting components such as lipids may interfere with detection accuracy or suppress ionization efficiency in mass spectrometric analysis. In addition, these matrix residues can accumulate in the chromatographic system, potentially compromising column performance and requiring more frequent maintenance.

In vegetable oils, tocopherols are predominantly present in their free form, which are readily extractable and possess strong antioxidant activity. However, a small proportion may exist in esterified forms, bound to fatty acids or other lipid components via ester linkages. To accurately quantify the total tocopherol content—including both free and bound fractions—saponification is commonly applied prior to analysis [36]. This procedure usually involves alkaline hydrolysis, typically using ethanolic potassium hydroxide or sodium hydroxide under controlled reflux conditions, to cleave ester bonds and release bound tocopherols [27]. To enhance hydrolysis efficiency, ultrasound-assisted saponification has been explored as an effective alternative. For example, Zhang et al. demonstrated that in tea seed and olive oils, ultrasound-assisted saponification achieved tocopherol recoveries ranging from 81.7% to 112.0% within 40 min, while preserving the integrity of thermolabile compounds [10]. However, the saponification process must be carefully optimized. An excessive alkaline concentration, elevated temperatures, or prolonged reaction times can lead to the oxidative degradation or structural isomerization of tocopherols, thereby compromising the quantification accuracy. Moreover, saponification may generate interfering byproducts or matrix effects, particularly in complex oil samples, which necessitates subsequent purification steps such as liquid–liquid extraction or solid-phase extraction prior to chromatographic analysis.

### 3.2. Solvent Extraction

Solvent extraction remains one of the most fundamental approaches for isolating tocopherols from vegetable oils, with conventional LLE being widely adopted due to its operational simplicity and cost-effectiveness [37]. These techniques exploit the differences in partition coefficients between tocopherols and the oil matrix, enabling their transfer into a suitable organic solvent [38]. An ideal solvent should exhibit high solubility for tocopherols while remaining immiscible with the oil phase. Commonly used solvents include methanol, ethanol, and acetonitrile [12,39]. Since the partition coefficient is a constant under defined conditions, these methods offer good reproducibility and are suitable for both laboratory-scale studies and certain industrial applications.

To enhance extraction efficiency, auxiliary techniques such as mechanical shaking, ultrasonic assistance, and continuous reflux (as in Soxhlet extraction) are often employed. For example, Soxhlet extraction using pure organic solvents in multiple cycles has been shown to improve the recovery of tocopherols making it particularly suitable for purification and preparative purposes. Özcan et al. reported that the α-tocopherol content in Moringa seed oil reached 151.67 mg/kg when extracted using a Soxhlet system. They also found that the levels of α-, γ-, and δ-tocopherols in Soxhlet-extracted oil were significantly higher than those obtained through cold-pressing methods [40].

Despite these advantages, solvent extraction methods present certain limitations. They typically require large volumes of organic solvents, involve lengthy extraction times, and may expose tocopherols to elevated temperatures for extended periods, potentially leading to the thermal degradation or oxidative loss of heat-sensitive compounds [41,42]. These drawbacks highlight the need for more efficient and environmentally friendly extraction strategies in tocopherol analysis.

### 3.3. Supercritical Fluid Extraction

Supercritical fluid extraction with carbon dioxide (SFE-CO_2_) has garnered considerable attention in natural product extraction due to its tunable selectivity, non-toxic nature, and suitability for isolating thermally labile compounds [43,44]. Supercritical CO_2_ exhibits gas-like diffusivity and liquid-like solvating power. By precisely adjusting the temperature and pressure, the selective enrichment of target analytes can be achieved [45].

SFE-CO_2_ has been successfully applied to extract tocopherols from various vegetable oils, including Hibiscus sabdariffa seed oil [15] and guava seed oil [46]. Peng et al. optimized the extraction of γ-tocopherol from Hibiscus seed oil under 30 MPa and 40 °C, obtaining a yield ranging from 1.6 to 5.6 mg of γ-tocopherol per 100 g of oil. Regression analysis revealed a strong correlation between process variables and extraction efficiency (R^2^ = 0.9754), highlighting the method’s controllability and reproducibility [15].

Despite its advantages, SFE presents certain challenges. High equipment costs and elevated operating pressures limit its routine application in standard laboratory settings [47]. Additionally, the extraction efficiency for moderately polar or esterified tocopherol derivatives is suboptimal [48]. Pre-treatment steps such as saponification or pre-purification are often required to enhance recovery.

### 3.4. Liquid-Phase Microextraction

Liquid-phase microextraction (LPME) has emerged as a miniaturized and environmentally friendly sample preparation technique, characterized by low solvent consumption, simplified operation, and high enrichment efficiency [49].

Conventional LPME formats, such as dispersive liquid–liquid microextraction (DLLME) and hollow-fiber LPME (HF-LPME), have been widely applied in the extraction of hydrophobic analytes from complex matrices [50,51]. These techniques rely on the dispersion of a small volume of organic solvent into the sample solution, forming fine droplets that facilitate rapid mass transfer [51]. While effective in reducing solvent usage and improving enrichment efficiency, traditional LPME systems often employ volatile or toxic solvents, limiting their suitability for routine tocopherol analysis in food-related applications. These limitations have prompted the search for greener and more selective extraction media.

Among various LPME strategies, the use of deep eutectic solvents (DESs) as extraction media has garnered increasing attention, particularly in the isolation of natural products [52,53]. Typically composed of hydrogen bond donors (HBDs) and acceptors (HBAs) in defined molar ratios, DESs form stable liquid-phase systems under mild conditions. Compared with conventional organic solvents, DESs offer distinct advantages in selectively extracting both polar and nonpolar compounds from complex matrices. Moreover, their composition can be flexibly tuned by adjusting the HBD and HBA components, which enhances their potential for extracting lipophilic bioactives from vegetable oils [54].

Xie et al. developed a DES composed of tetrabutylammonium chloride (TBAC) and ethanol and applied it in an LPME mode to simultaneously extract α-, β-, γ-, and δ-tocopherols from various vegetable oils. This method achieved high sensitivity (limits of detection of 2.1–3.0 ng/mL) and excellent recovery rates (80.7–105.4%), while significantly reducing the use of traditional organic solvents—demonstrating strong applicability and eco-friendliness [55].

Chen et al. introduced a phenolic DES composed of TBAC and 4-methylphenol in a 1:1 molar ratio for the rapid and efficient extraction of α-, γ-, and δ-tocopherols from soybean oil deodorizer distillate fatty acid methyl esters (SODD-MEs). The method operates under ambient conditions and requires only 1 min of vortexing, using minimal solvent volumes. Under optimized conditions (DES/SODD-ME mass ratio of 6:1 and n-hexane/SODD-ME ratio of 1:1), the total extraction efficiency reached 97.5%. Mechanistic studies suggest that hydrogen bonding and π–π interactions between tocopherols and the phenolic DES components synergistically enhance binding affinity and selectivity [56].

### 3.5. Solid-Phase Extraction

**Figure 3:**
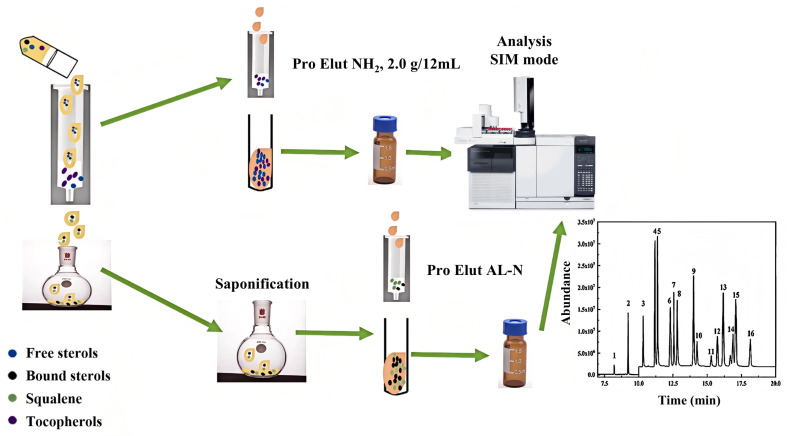
Schematic diagram of the determination of tocopherols in vegetable oils using ProElut NH_2_ SPE cartridges (1.0 g/6 mL) combined with saponification and GC-MS [13].

SPE has been widely adopted as a robust and efficient sample pretreatment technique for the enrichment and purification of lipophilic micronutrients, such as tocopherols, in vegetable oils. Traditional SPE protocols, commonly utilizing silica-based sorbents, have demonstrated the effective separation of α-tocopherol and squalene from olive oil [57]. In addition, other commercial sorbents—such as ProElut NH_2_ SPE cartridges (1.0 g/6 mL)—have been employed for the simultaneous extraction and concentration of free phytosterols and tocopherols from vegetable oils. These were followed by trimethylsilyl (TMS) derivatization and subsequent analysis via GC-FID, achieving spiked recovery rates between 83.4% and 97.7% (Figure 3) [14].

To improve selectivity and adaptability to complex matrices, recent research has shifted toward the development of advanced sorbent materials. For instance, resin-based directional polymers (RDPs), known for their high specificity and cost-effectiveness, have been utilized as novel SPE sorbents for the efficient extraction of α-tocopherol and other lipid-soluble compounds from complex samples such as sunflower oil. These systems significantly reduce solvent consumption and streamline sample preparation, demonstrating strong potential for industrial-scale applications [58]. Kalogiouri et al. employed sol-gel-coated polyester membranes as sorptive media for tocopherol extraction. This approach markedly enhanced the extraction efficiency of α-, β + γ-, and δ-tocopherols, achieving low detection limits (as low as 0.05 μg/g) and high recoveries ranging from 88.7% to 95.1%, with excellent reproducibility—making it particularly suitable for trace-level analysis in lipid-rich matrices [59]. Modified ZSM-5 zeolites, prepared via desilication under alkaline conditions, exhibit hierarchical porosity and enhanced mesostructures that improve the adsorption capacity and selectivity for α-tocopherol and β-sitosterol. When applied to deodorizer distillates of sunflower oil, this material achieved outstanding recovery rates—99.20% for α-tocopherol and 97.32% for β-sitosterol—far surpassing unmodified materials [60].

**Figure 4:**
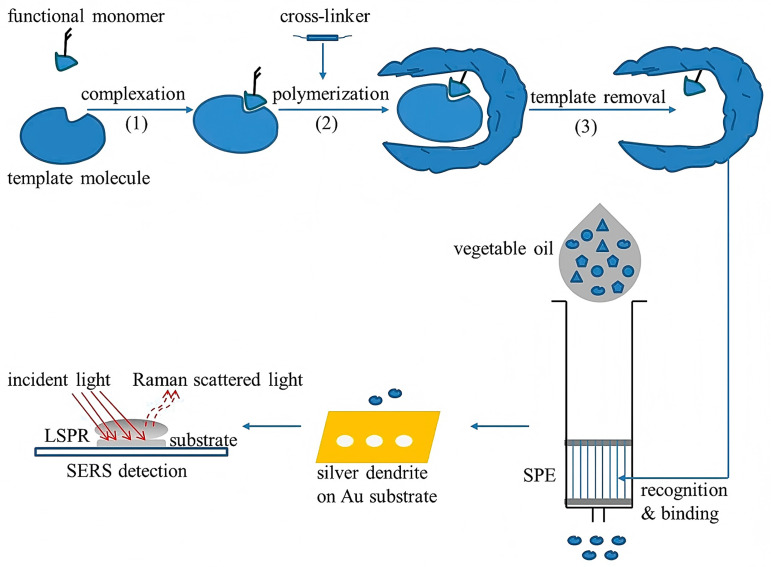
Schematic illustration of the molecularly imprinted polymers–surface-enhanced Raman spectroscopy (MIPs-SERS) biosensing system for the detection of α-tocopherol in vegetable oils. Methacrylic acid was used as the functional monomer, with ethylene glycol dimethacrylate as the cross-linker [61].

Molecularly imprinted solid-phase extraction (MIP-SPE), owing to its excellent selectivity, has also been applied for the targeted enrichment of α-tocopherol in vegetable oils. Feng et al. synthesized molecularly imprinted polymers (MIPs) using α-tocopherol as the template and packed them into SPE cartridges for the selective extraction of α-tocopherol from hexane-diluted oil samples (Figure 4) [61]. Elution was carried out using ethanol containing 5% acetic acid, and the eluate was subsequently analyzed via surface-enhanced Raman spectroscopy (SERS) employing dendritic silver nanostructures. This MIP-SERS system enabled the rapid (≤15 min), sensitive, and selective detection of α-tocopherol, showing excellent model performance (R > 0.92, RMSE < 0.41) across various oil types.

Overall, the integration of novel functional materials and microstructured sorbents is transforming SPE into a highly selective, sensitive, and environmentally friendly platform. These advances are accelerating the development of more automated and green analytical pipelines for tocopherol quantification in complex vegetable oil matrices, with significant implications for natural product analysis and quality control.

### 3.6. Magnetic Solid-Phase Extraction

Magnetic solid-phase extraction (MSPE) is a sample preparation technique that integrates the high selectivity of traditional solid-phase extraction (SPE) with the rapid and controllable separation capabilities of magnetic nanomaterials [62]. By employing an external magnetic field, magnetic sorbents can be rapidly enriched and separated, simplifying the extraction process [63]. In a representative study, an MSPE-GC-MS method based on magnetic graphene oxide (MGO) was developed for the simultaneous extraction and analysis of α-tocopherol and β-sitosterol from soybean oil and rapeseed oil deodorizer distillates (SODDs and RODDs). Key parameters—such as the type and volume of desorption solvent, the amount of MGO, and the desorption time—were systematically optimized. Under optimal conditions, the average recovery of α-tocopherol reached 94.44% [64]. This study highlights MSPE as a simple, efficient, and highly sensitive technique for the rapid extraction of lipophilic compounds from complex oil-based matrices. Despite its promise, the application of MSPE in tocopherol extraction from vegetable oils remains relatively limited. Given the availability of a wide range of functionalized magnetic sorbents, future research may focus on integrating tailored magnetic materials to broaden the scope of MSPE for analyzing the bioactive components in vegetable oils.

In summary, extraction techniques for tocopherols from vegetable oils are rapidly evolving toward greener, more efficient, and automation-friendly methodologies. While traditional solvent extraction remains the foundational approach due to its operational maturity and wide applicability, its environmental limitations are becoming increasingly apparent. Supercritical fluid extraction offers superior selectivity and environmental advantages but is constrained by high equipment costs and complex operations. Solid-phase extraction and its derivatives demonstrate notable benefits in terms of selectivity, scalability, and integration with automated systems—especially for trace-level enrichment in complex matrices. Most notably, DES-based microextraction techniques represent a promising frontier, striking a balance between environmental sustainability, extraction efficiency, and operational simplicity. Their rapid development may redefine future protocols for tocopherol analysis in both research and industry.

## 4. Analytical Methods for the Tocopherols in Vegetable Oils

After sample preparation, the selection of appropriate analytical techniques is essential to ensure the accurate quantification and characterization of tocopherol components in vegetable oils. A range of methods has been developed, encompassing chromatographic, spectroscopic, mass spectrometry-based, and electrochemical techniques [39,65]. Table 1 summarizes representative analytical approaches, detailing sample preparation strategies, detection modes, chromatographic configurations, mobile phase compositions, and detection limits. This overview provides a practical reference for evaluating the strengths and applicability of each method within different analytical scenarios.

### 4.1. Chromatographic Techniques

Both reversed-phase and normal-phase modes are widely employed in high-performance liquid chromatography (HPLC) for tocopherol analysis, with the choice determined based on the polarity characteristics of the stationary phase and mobile phase. In reversed-phase systems, C18 columns combined with methanol- or acetonitrile-based eluents enable the efficient separation of tocopherol isomers, making RP-HPLC the most commonly used approach [15]. In contrast, normal-phase systems using silica-based columns—such as Phenomenex Luna Silica (150 × 4.6 mm, 3 μm) with n-hexane/ethyl acetate [33] and Ascentis Si (250 × 4.6 mm, 5 μm) with n-hexane/isopropanol (99:1, *v*/*v*) [39]—are preferred in non-aqueous environments or when specific selectivity is required.

Early studies primarily focused on the rapid and sensitive quantification of individual tocopherols. For example, a method utilizing a C18 column with a methanol/tetrahydrofuran (90:10, *v*/*v*) mobile phase and fluorescence detection allowed efficient separation of α-, β-, γ-, and δ-tocopherols, achieving detection limits as low as 6–7 ng/g and excellent repeatability (CV < 2.8%) across various vegetable oil matrices [28]. As research interest in lipid-soluble bioactives has grown, HPLC methods have evolved toward multi-analyte detection. A representative study demonstrated the simultaneous quantification of tocopherols, phytosterols, and squalene using a methanol–water/isopropanol system with UV detection at 210 nm, significantly improving the analytical throughput and enabling the comprehensive profiling of functional components in vegetable oils [27]. Dual-mode detection systems combining UV (290 nm) and fluorescence (296/398 nm) have also been introduced to address matrix complexity, enabling the rapid quantification of α-, γ-, and δ-tocopherols alongside coexisting retinol and esterified compounds. These methods have achieved analysis times under 10 min, making them well-suited for high-throughput applications [8]. Recent advancements have further refined chromatographic conditions and detection strategies. Notably, the use of ultra-high performance and core–shell columns with enhanced column efficiency has expanded the analytical scope from single-analyte determination to multiplexed quantification in complex lipid matrices—delivering faster and more robust analysis.

HPLC has proven particularly valuable in the detection of adulteration and authentication of vegetable oils. For instance, the α/(β + γ) tocopherol ratio has been proposed as a discriminatory marker to differentiate extra virgin olive oil (EVOO) from adulterated samples containing sunflower or peanut oil [28,66,67]. In another study, α-tocopherol served as a marker compound for detecting sunflower oil adulteration in EVOO using a non-saponification RP-HPLC method with fluorescence detection. Adulteration levels as low as 5% were successfully identified, demonstrating the method’s high sensitivity and practical utility in “oil-in-oil” fraud detection [68].

These developments underscore the versatility and analytical strength of HPLC in tocopherol determination—advancing not only nutritional evaluations but also contributing significantly to food authenticity, safety, and traceability. Future research is expected to further integrate HPLC with hyphenated detection systems and automation, paving the way for more comprehensive and scalable solutions in the authentication and quality assessment of edible oils.

### 4.2. Capillary Electrophoresis

Capillary electrophoresis (CE) has been investigated as a promising analytical technique for the determination of tocopherols in vegetable oils due to its high separation efficiency, low sample and solvent consumption, and environmentally friendly profile [69]. This technique operates based on the differential electrophoretic mobility of analytes under an electric field, making it particularly suitable for the rapid analysis of small, structurally similar compounds such as tocopherol isomers [70].

In a notable study, Galeano-Díaz et al. established and optimized a CE method for tocopherol determination in vegetable oils by employing experimental design and response surface methodology. The separation was achieved using a methanolic background electrolyte composed of 12 mM borate (including 3 mM tetraborate), 60 mM sodium cholate, and 12 mM sodium hydroxide. Hydrodynamic injection was used for sample introduction, and tocopherols were detected using both UV and fluorescence detectors. The fluorescence mode offered enhanced sensitivity, with quantification limits ranging from 0.18 to 0.56 μg/mL for different tocopherol isomers [71].

Despite its methodological advantages, including high efficiency, miniaturization potential, and low environmental impact, CE remains underutilized in the routine tocopherol analysis of vegetable oils. Its limited use is mainly attributed to low sensitivity (due to small injection volumes), poor compatibility with complex matrices, and limited reproducibility of migration times. Nevertheless, further advances in capillary surface modification, background electrolyte optimization, and on-line sample preconcentration techniques may help to overcome current limitations. These developments could expand the applicability of CE in the trace-level determination of lipophilic micronutrients within complex lipid matrices, offering a viable and greener alternative to more established analytical platforms.

### 4.3. Mass Spectrometry-Based Hyphenated Techniques

**Figure 5:**
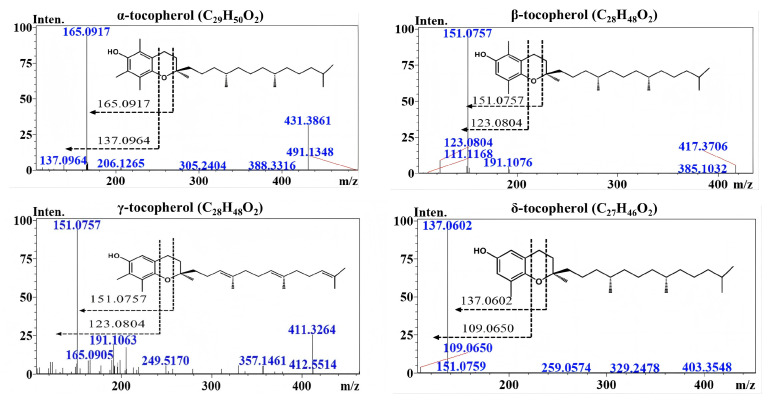
MS/MS spectra of α-, β-, γ-, and δ-tocopherols [35].

LC-MS/MS has been applied to the determination of tocopherols in vegetable oils due to its high sensitivity and strong tolerance to complex matrices [72]. Compared with conventional UV or fluorescence detectors, MS-based techniques effectively address issues such as co-elution and peak overlap, making them particularly suitable for the accurate quantification of multiple analytes in complex lipid matrices [9,20,73]. For instance, Zhang et al. developed an LC-MS/MS method using a methanol–water mobile phase and multiple reaction monitoring (MRM) mode for the simultaneous determination of tocopherols, carotenoids, and phytosterols in vegetable oils. The method achieved limits of detection ranging from 2.0 to 3.2 ng/mL and quantification limits between 6.1 and 10.0 ng/mL [10]. Specifically, α-, β-, γ-, and δ-tocopherols exhibit [M + H]^+^ ions at m/z 431.38, 417.37, 417.37, and 403.35, respectively [35]. Figure 5 displays their corresponding MS/MS spectra of tocopherols.

To further enhance analytical throughput and promote green chemistry principles, supercritical fluid chromatography coupled with mass spectrometry (SFC-MS) has been explored for tocopherol analysis. Using CO_2_ and ethanol as the mobile phase, in combination with an NH_2_ stationary phase and atmospheric pressure photoionization (APPI) source, the method achieved analysis times of less than 5 min while significantly reducing organic solvent consumption, all without compromising sensitivity [34].

In addition to liquid chromatography systems, gas chromatography–mass spectrometry (GC-MS) has also been employed for the simultaneous analysis of tocopherols and their homologues [74]. Tocopherols exhibit inherently low volatility, necessitating derivatization—most commonly with trimethylsilyl (TMS) reagents—prior to gas chromatographic analysis [75,76]. Zhang et al. demonstrated that this approach enables the separation and quantification of α- to δ-tocopherols, as well as various tocotrienols, within 14 min, achieving detection limits as low as 0.3 ng/mL and offering excellent resolution and quantification accuracy [12].

Collectively, LC-MS/MS, SFC-MS, and GC-MS provide powerful and versatile platforms for the rapid and reliable determination of tocopherol components in complex vegetable oil matrices. These hyphenated techniques not only enhance analytical performance but also support the development of robust methodologies for profiling bioactive lipid-soluble micronutrients, making them the current mainstream and routine technologies for tocopherol analysis.

### 4.4. Spectroscopic Techniques

Due to the structural characteristics of tocopherols—including phenolic hydroxyl groups (–OH), aromatic rings, and long-chain alkyl side chains—these compounds exhibit distinct molecular vibrations and electronic transitions. These features render them highly amenable to spectroscopic techniques such as near-infrared (NIR), Fourier-transform infrared (FT-IR), and Raman spectroscopy [77]. By capturing spectral signatures associated with molecular absorption or scattering in response to specific wavelengths, these methods allow for the indirect quantification of tocopherols. As rapid, non-destructive, and cost-effective analytical approaches, spectroscopic techniques are increasingly recognized as valuable complements to chromatographic methods, especially in scenarios requiring high-throughput screening or real-time monitoring.

#### 4.4.1. Near-Infrared Spectroscopy

NIRS detects overtone and combination bands associated with molecular vibrations, particularly those involving –CH, –OH, and –NH functional groups [78,79]. In tocopherol analysis, its sensitivity to alkyl chains and hydroxyl functionalities has facilitated its application in both quantitative analysis and sample classification. For instance, Cayuela and García employed NIRS coupled with partial least squares (PLS) regression to rapidly quantify α-tocopherol and total tocopherol contents in olive oil. The resulting calibration models exhibited strong predictive power, with residual predictive deviations (RPDs) of 2.37 and 2.01 [80], respectively. Additionally, when combined with PLS-discriminant analysis (PLS-DA), the model achieved high specificity (0.96) and sensitivity (0.84) in classifying high α-tocopherol samples, reaching nearly 100% classification accuracy. In another study, a PLS model constructed using the 6500–4500 cm^−1^ spectral range enabled the accurate quantification of standard α-tocopherol solutions and vegetable oil samples, reinforcing the potential of NIRS for routine tocopherol screening [16]. These findings highlight NIRS as a promising platform for the rapid quality control of vitamin E-rich oils, particularly in industrial settings where minimal sample preparation is preferred.

#### 4.4.2. Fourier-Transform Infrared Spectroscopy

Unlike NIRS, which primarily captures overtones and combination vibrations, FT-IR targets fundamental molecular vibrations, providing more specific information on characteristic functional groups [81]. By analyzing spectral features in the 1472–1078 cm^−1^ range, Silva et al. developed a PLS-based calibration model to predict the α-tocopherol content in 13 types of vegetable oils without any sample pretreatment [82]. The results were statistically comparable to those obtained via HPLC-fluorescence detection, demonstrating the accuracy and operational simplicity of the method. Moreover, Ahmed et al. integrated FT-IR spectral data with multivariate analysis to estimate the total content of tocopherols, tocotrienols, and plastochromanol-8 across several oil types, including canola, flaxseed, soybean, and sunflower oils [83]. This approach not only accelerated analysis but also provided a holistic assessment of vitamin E compounds, offering valuable insights for quality assurance and the nutritional profiling of vegetable oils. These results underscore FT-IR as an efficient and scalable solution for comprehensive vitamin E monitoring in lipid matrices.

#### 4.4.3. Raman Spectroscopy

**Figure 6:**
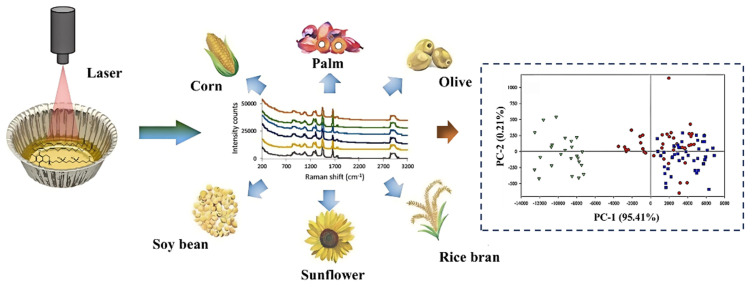
Classification of vegetable oils based on chemometrics and Raman spectroscopy [86].

Raman spectroscopy relies on the inelastic scattering of monochromatic light and is particularly sensitive to asymmetric stretches and conjugated ring systems, making it suited for the detection of aromatic compounds such as α-tocopherol [84,85]. In a recent study, Moe Htet et al. combined Raman spectroscopy with PLS regression to quantify α-tocopherol across six vegetable oils without requiring sample pretreatment (Figure 6) [86]. The resulting model demonstrated strong predictive performance (R^2^ > 0.95), and the application of orthogonal signal correction significantly enhanced model robustness and accuracy.

### 4.5. Electrochemical Methods

Electrochemical techniques have emerged as powerful tools for the detection of tocopherols in vegetable oils due to their high sensitivity, rapid response, low instrumentation cost, and minimal sample preparation requirements [87,88,89]. Recent advances in this field have focused on the application of voltammetric methods, the functional modification of electrode materials, and the development of multimodal sensing platforms.

Among the basic techniques, differential pulse voltammetry (DPV) and square wave voltammetry (SWV) have been widely applied for the quantification of tocopherols in oil matrices. By optimizing electrode configurations and extraction conditions—such as using carbon paste electrodes with silicone oil-based pretreatment in 0.1 M nitric acid medium—the efficient preconcentration of tocopherols was achieved, thereby enhancing analytical sensitivity. Under enrichment times of 5–15 min, the detection limits for α-tocopherol reached 1.0 × 10^−7^ and 3.3 × 10^−9^ mol·L^−1^, respectively, with good linearity and accuracy comparable to that of HPLC methods [90]. Moreover, through fine-tuning of the organic solvent system (e.g., hexane–ethanol mixtures) and electrochemical parameters, the partial resolution of oxidation peaks corresponding to α-, γ-, and δ-tocopherol was achieved, suggesting the feasibility of simultaneous multi-analyte detection [91].

To further enhance electrochemical sensitivity and selectivity, considerable efforts have been dedicated to the functionalization of electrode materials. For instance, a Nafion/graphene nanocomposite-modified glassy carbon electrode exhibited excellent electrocatalytic activity for α-tocopherol oxidation in aqueous–organic media, achieving a detection limit as low as 0.06 μmol·L^−1^ with high reproducibility and resistance to interference. Satisfactory recoveries were obtained in real-world samples, such as vegetable oils and fortified beverages [92]. In addition, the miniaturization of electrochemical setups using pencil graphite electrodes (PGEs) has demonstrated potential for the rapid screening of oil authenticity. A current-response-based method enabled the detection of adulteration in oil samples, with a detection limit of 10% (*w*/*w*) adulteration—offering a promising approach for portable authenticity assessments [93]. Another approach involved coupling an electrochemical detection unit with a supercritical fluid chromatography system using a CO_2_–methanol–ammonium acetate mobile phase. This allowed the simultaneous separation and picomolar-level quantification of α-, β-, γ-, and δ-tocopherols, with excellent repeatability and signal stability [94].

Collectively, these advancements indicate a promising trajectory for electrochemical sensors in the rapid, sensitive, and multiplexed analysis of tocopherols in complex oil matrices. Future research should continue to explore miniaturization, integration with smart sensing materials, and data-driven chemometric modeling to achieve fully autonomous and field-deployable detection platforms.

## 5. Conclusions

Tocopherols, as essential components of vitamin E, consist of several isomers with distinct distribution patterns across various vegetable oils, emphasizing the need for accurate and efficient analytical methods. Recent advancements in extraction techniques, such as SPME and LLE, have significantly enhanced sample preparation by improving selectivity and compatibility with complex oil matrices. However, challenges persist, including the reliance on solvents and the time-consuming nature of traditional methods, which may hinder both efficiency and sustainability. The dependence on organic solvents, in particular, raises concerns regarding their environmental impact, highlighting the need for greener alternatives.

Analytical techniques, ranging from chromatographic separations and mass spectrometry to spectroscopic and electrochemical sensors, offer complementary advantages in terms of sensitivity, specificity, and throughput. Despite these advancements, matrix interferences continue to pose significant challenges. Emerging solutions, such as MIPs for selective extraction and machine learning algorithms for interference correction, offer promising avenues to address these issues.

Beyond their application in quality control, tocopherol analysis has broader practical implications, including nutritional labeling and adulteration detection, underscoring the importance of accurate and reliable analytical strategies in real-world applications. Looking ahead, the integration of rapid sensors and AI-driven analytics is expected to play a pivotal role in future quality control workflows, facilitating more efficient and scalable detection platforms.

## Figures and Tables

**Table 1:** The advanced methods used for tocopherols in vegetable oils.

Sample	Sample Preparation	Adsorbent/Solvent	Analytical Method	Analytical Column	Mobile Phase	Recovery, %	LOD	Reference
8 kinds of oils	Organic solvent dilution	Diluted in acetonitrile/tetrahydrofuran	HPLC-FLD	HyPurity C18 column (250 × 4.6 mm i.d., 5 μm)	Tetrahydrofuran and methanol (10:90, *v*/*v*)	98.8–102.0	6–7 ng/g	[28]
Canola oil	Organic solvent dilution	Diluted in hexane	HPLC-DAD-MS/MS	Phenomenex Luna silica column (150 × 4.6 mm, i.d., 3 μm)	n-Hexane and Ethyl acetate, gradient elution	90.9–107	0.02–0.10 µg/mL	[33]
5 kinds of oils	Organic solvent dilution	Diluted in hexane	HPLC-UV	Lichrosorb RP-18 column (150 × 4.0 mm i.d., 10 μm)	Hexadecyltrimethylammonium bromide (0.1 M) and n-propanol (65%, *v*/*v*)	/	0.05–0.09 µg/mL	[8]
Walnut oil	Organic solvent dilution	Diluted in hexane	SFC-QTOF-MS	Thermo Scientific BEH 2-Ethylpyridine (2-EP) column (100 × 3.0 mm i.d., 1.7 μm)	CO_2_ and methanol containing 0.1% formic acid, gradient elution	70.61−101.44	0.05−0.20 ng/mL	[35]
Soybean oil	Organic solvent dilution	Diluted in hexane	SFC-MS	Six different analytical columns were compared to optimize condition	CO_2_ and ethanol with 0.1% formic acid, gradient elution	84.97−100.33	0.025−40.27 ng/mL	[34]
4 kinds of oils	Saponification	NaOH	HPLC-DAD	ZORBAX Eclipse Plus C18 column (250 mm × 4.6 mm, 5 μm)	Methanol and water and isopropanol (98:2, *v*/*v*)	91.2–102.2	0.10–0.25 µg/mL	[27]
Camellia oils and olive oils	Ultrasound-assisted saponification	At temperature of 75 °C for 40 min.	LC-MS/MS	Thermo Scientific C18 column (100 × 2.1 mm i.d., 3 μm)	Methanol (0.02% aqueous formic acid) and acetonitrile (95:5, *v*/*v*)	90.3−112.0	2.0–3.2 ng/mL	[10]
8 kinds of oils	UAE- LLE	Methanol	GC-MS	DB-5MS capillary column (30 m × 0.25 mm, 0.25 μm)	Helium (99.999% purity)	83.7−117.2	0.3−2.5 ng/mL	[12]
Olive Oils	UAE- LLE	Methanol/water (3:2, *v*/*v*)	HPLC-FID	Ascentis Si column (250 × 4.6 mm i.d., 5 μm)	n-hexane and isopropanol (99:1, *v*/*v*)	80.1−90.6	1−2 ng/mL	[39]
9 kinds of oils	GPC	Cyclohexane/ethyl acetate (1:1, *v*/*v*)	HPLC-MS	YMC Carotenoid C30 column (250 × 4.6 mm, 5 μm)	Methanol and water (98:2, *v*/*v*)	81.5−113.8	1 µg/g	[9]
15 kinds of oils	SPE	Sep-Pak cartridge (Pro Elut NH2, 2.0 g/12 mL)	GC-MS	DB-5 MS capillary column (30 m × 0.25 mm, 0.25 μm)	Helium (99.999% purity)	91.29−111.93	0.010–0.050 µg/mL	[13]
5 kinds of oils	SPE	Sep-Pak cartridge	GC-FID	DB-5MS capillary column (30 m × 0.25 mm i.d., 0.25 μm)	Helium (99.999% purity)	83.4−97.7	0.5 µg/g	[14]
5 kinds of oils	FPSE	Sol-gel polycaprolactone-polydimethylsiloxane-polycaprolactone coated polyester FPSE membrane	HPLC-DAD	Nucleosil C18 column (250 mm × 4.6 mm, 5 μm)	MeOH and ACN, gradient elution	90.8−95.1	0.05–0.10 µg/g	[59]
Soybean and rapeseed oil deodorizer distillate	MSPE	MGO	GC-MS	HP-5MS capillary column (30 m × 0.25 mm i.d., 0.25 μm)	Helium (99.999% purity)	94.44−97.77	0.05–0.01 mg/g	[64]

FPSE: fabric phase sorptive extraction; GPC: gel permeation chromatography; MGO: magnetic graphene oxide.

## Data Availability

No new data were created or analyzed in this study. Data sharing is not applicable to this article.

## Abbreviations

The following abbreviations are used in this manuscript:

APPI	Atmospheric pressure photoionization
CE	Capillary electrophoresis
DESs	Deep eutectic solvents
DLLME	Dispersive liquid–liquid microextraction
DPV	Differential pulse voltammetry
EVOO	Extra virgin olive oil
FT-IR	Fourier-transform infrared spectroscopy
GC-MS	Gas chromatography coupled with mass spectrometry
HBA	Hydrogen bond acceptor
HBD	Hydrogen bond donor
HF-LPME	Hollow-fiber liquid-phase microextraction
HPLC	High-performance liquid chromatography
HPLC-MS	High-performance liquid chromatography coupled with mass spectrometry
HPLC-UV	High-performance liquid chromatography with ultraviolet detection
LC-MS/MS	Liquid chromatography coupled with tandem mass spectrometry
LLE	Liquid–liquid extraction
LPME	Liquid-phase microextraction
MGO	Magnetic graphene oxide
MIP-SPE	Molecularly imprinted solid-phase extraction
MIPs	Molecularly imprinted polymers
MRM	Multiple reaction monitoring
MSPE	Magnetic solid-phase extraction
NIR	Near-infrared spectroscopy
PGE	Pencil graphite electrode
PLS	Partial least squares
PLS-DA	Partial least squares–discriminant analysis
RDPs	Resin-based directional polymers
RODD	Rapeseed oil deodorizer distillate
ROS	Reactive oxygen species
RPDs	Residual predictive deviations
SERS	Surface-enhanced Raman spectroscopy
SFC-MS	Supercritical fluid chromatography coupled with mass spectrometry
SFE-CO_2_	Supercritical fluid extraction with carbon dioxide
SODD	Soybean oil deodorizer distillate
SODD-ME	Soybean oil deodorizer distillate fatty acid methyl esters
SPE	Solid-phase extraction
SWV	Square wave voltammetry
TBAC	Tetrabutylammonium chloride
TMS	Trimethylsilyl
UV	Ultraviolet
